# Baicalin-aluminum alleviates necrotic enteritis in broiler chickens by inhibiting virulence factors expression of *Clostridium perfringens*

**DOI:** 10.3389/fcimb.2023.1243819

**Published:** 2023-09-25

**Authors:** Jin Liu, Shuangqi Wu, Honghao Zhao, Chun Ye, Shulin Fu, Yu Liu, Ting Liu, Yinsheng Qiu

**Affiliations:** ^1^ Hubei Key Laboratory of Animal Nutrition and Feed Science, Wuhan Polytechnic University, Wuhan, China; ^2^ Hubei Collaborative Innovation Center for Animal Nutrition and Feed Safety, Wuhan Polytechnic University, Wuhan, China

**Keywords:** *Clostridium perfringens*, baicalin-aluminum, necrotic enteritis, virulence factors, broiler chickens

## Abstract

*Clostridium perfringens* type A is the main cause of necrotic enteritis (NE) in chickens. Since the use of antibiotics in feed is withdrawn, it is imperative to find out suitable alternatives to control NE. Baicalin-aluminum complex is synthesized from baicalin, a flavonoid isolated from *Scutellaria baicalensis* Georgi. The present study investigated the effects of baicalin-aluminum on the virulence-associated traits and virulence genes expression of *C. perfringens* CVCC2030, it also evaluated the *in vivo* therapeutic effect on NE. The results showed that baicalin-aluminum inhibited bacterial hemolytic activity, diminished biofilm formation, attenuated cytotoxicity to Caco-2 cells, downregulated the expression of genes encoding for clostridial toxins and extracellular enzymes such as alpha toxin (CPA), perfringolysin O (PFO), collagenase (ColA), and sialidases (NanI, NanJ). Additionally, baicalin-aluminum was found to negatively regulate the expression of genes involved in quorum sensing (QS) communication, including genes of Agr QS system (*agrB*, *agrD*) and genes of VirS/R two-component regulatory system (*virS*, *virR*). *In vivo* experiments, baicalin-aluminum lightened the intestinal lesions and histological damage, it inhibited pro-inflammatory cytokines (TNF-α, IL-1β, IL-6) expression in the jejunal and ileal tissues. Besides, baicalin-aluminum alleviated the upregulation of *C. perfringens* and *Escherichia coli* and raised the relative abundance of *Lactobacillus* in the ileal digesta. This study suggests that baicalin-aluminum may be a potential candidate against *C. perfringens* infection by inhibiting the virulence-associated traits and virulence genes expression.

## Introduction

Enteric diseases are of major concern to the poultry industry due to the increased mortality, impaired production and potential contamination of poultry products. Necrotic enteritis (NE) caused by *Clostridium perfringens* is considered one of the most common enteric diseases, causing huge economic losses to the global poultry industry (Wade et al., 2015). Generally, the prevention and treatment depend on the usage of antibiotics about NE. Extensive use of antibiotics gives rise to the generation and spread of antibiotic-resistant pathogens and their resistance genes to humans, animals, and environment in global animal production, becoming a major threat to animal food safety and human health. Currently, the use of antibiotics as a growth promoter has been prohibited or restricted worldwide in animal feeds, resulting in a significant increasing of NE outbreaks across the poultry industry and serious economic losses. Thereby, seeking for effective and environment-friendly alternatives to counter bacterial infections are becoming increasingly important. Notably, attenuating the mechanisms of bacterial virulence seems to be a promising alternative therapeutic strategy to overcome the problem of drug resistance.

*C. perfringens* is a gram-positive, anaerobic bacterium that is widely found in the water, soil, dust, faeces and normal gut microbiota of humans and animals. *C. perfringens* is responsible for the outbreaks of diverse diseases in humans and animals, including gas gangrene, gastroenteritis in humans, and NE in chickens (Kiu and Hall, 2018). Historically, *C. perfringens* isolates are classified into five pathogenic types (A thru E) depending on the production of typing toxins alpha (CPA), beta (CPB), epsilon (Etx), and iota (Itx) (Rood et al., 2018). For example, *C. perfringens* type A produce CPA, whereas type B strains produce CPA, CPB and Etx. *C. perfringens* type A strains are the main causative agents of NE in chickens. The pathogenicity is mainly mediated by secreting various extracellular toxins and enzymes, such as B-like toxin (NetB), CPA, perfringolysin O (PFO), collagenase (ColA) and sialidases, which play irreplaceable roles in the cytotoxicity to host cells and the development of NE in chickens (Mehdizadeh Gohari et al., 2021; Fathima and Hakeem, 2022). In addition, virulence-associated traits such as adhesion, gliding motility and biofilm formation should also be conducive to the survival and integrated virulence mechanism of *C. perfringens* (Valeriani et al., 2020).

Usually, regulation of virulence genes expression and virulence-associated traits in many bacteria relies on quorum-sensing (QS) system, which is a chemical communication process via population density-induced autoinducer signaling molecules to orchestrate group behaviors (Eickhoff and Bassler, 2018). The accessory gene regulator (Agr) QS system in *C. perfringens* is proved to regulate the production of key protein toxins and extracellular enzymes. The QS system is composed of a AgrD peptide and a AgrB transmembrane protein that processes the AgrD propeptide to an active autoinducing peptide (AIP) and transported from the cell. The AIP interacts with a VirS/R two-component regulatory system (TCRS) consisting of a VirS membrane sensor histidine kinase and a VirR response regulator to induce the autophosphorylation of VirS and subsequent phosphorylation of VirR (Li et al., 2020). Phosphorylated VirR can directly or indirectly regulate the expression of some *C. perfringens* toxin genes, such as *netB*, *pfoA*, *cpa*, *colA*, et al. (Ohtani and Shimizu, 2016). Apparently, the Agr QS system and VirS/R TCRS coordinately regulate the virulence genes expression and are critical for the pathogenesis of NE caused by *C. perfringens* in poultry (Yu et al., 2017).

A number of studies indicated that some plant-derived bioactive compounds were able to control bacterial infection via targeting virulence factors and slow down the rise of resistant strains (Liu et al., 2020; Hou et al., 2023; Martínez et al., 2023). Baicalin-aluminum is a complex of aluminum and baicalin. Baicalin is a flavonoid monomer purified from the *Scutellaria baicalensis* Georgi (Lamiaceae) root, it has been believed to possess broad pharmacological activities, such as anti-inflammatory, antitumor, antiviral and antibacterial activities (Xu et al., 2020). Particularly, baicalin may potentially be used to control bacterial infection by interfering with the QS system, down-regulating the expression of virulence factors, or inhibiting biofilm formation (Zhang et al., 2020; Zhang et al., 2021; Wang et al., 2023). Additionally, natural drugs can chelate with metal ions to form stable metallo-complexes, which have been proved to possess enhanced pharmacological effects as compared with the natural drugs alone (Gholami et al., 2020; Kondori et al., 2021). Our previous study demonstrated that baicalin-aluminum had good effect on the treatment of piglet diarrhea (Fu et al., 2019) and shaped the composition of gut microbiota in broiler chickens (Guo et al., 2021), suggesting that baicalin-aluminum might play a role in controlling enteric diseases in poultry. Nevertheless, scientific evidence is still lacking regarding the effects of baicalin-aluminum on the virulence factors expression and virulence phenotypes of *C. perfringens* and whether baicalin-aluminum has the potential to be developed as a new anti-virulence drug against NE.

In this study, we first examined the effects of baicalin-aluminum on the *in vitro* growth, hemolytic activity, biofilm formation, cytotoxicity and virulence genes expression of *C. perfringens*, and evaluated the therapeutic protection of baicalin-aluminum in an experimental chicken model of necrotic enteritis. This study indicates the possibility and importance of baicalin-aluminum as a potential novel agent for the prevention and treatment of *C. perfringens* infection.

## Materials and methods

### Bacterial strains and chemicals

**Figure 1:**
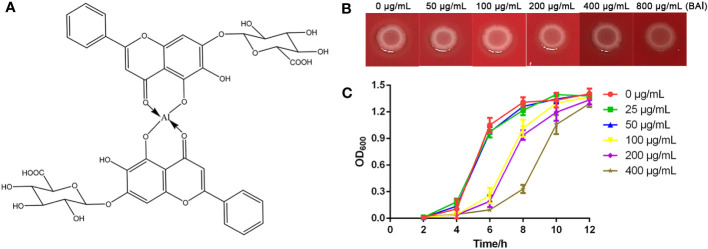
Antibacterial activity of baicalin-aluminum against *C. perfringens*. **(A)** Chemical structure of baicalin-aluminum. **(B)** MIC determination of baicalin-aluminum against *C. perfringens* CVCC2030. The colony formation on agar plates was used to assess the MIC_100_ value. **(C)** Growth curves of the *C. perfringens* treated with baicalin-aluminum (BAl). *C. perfringens* were treated with baicalin-aluminum (ranging from 0 to 400 µg/mL) under anaerobic conditions for 12 h. The absorbance of cultures was measured at OD_600_ every two hours to estimate the effect of baicalin-aluminum on the growth of *C. perfringens*. Data are expressed as means ± SD from three biological replicates.

Baicalin-aluminum (**Figure 1A**) was synthesized as previously described (Fu et al., 2019). Baicalin-aluminum was dissolved in dimethyl sulfoxide (DMSO) (Sigma Aldrich, St. Louis, MO, USA).

*C. perfringens* CVCC2030 (type A strain) was commercially obtained from the China Veterinary Culture Collection Center (CVCC). Media for culturing *C. perfringens* included TGY broth containing 3% tryptic soy broth (BD Difco, Sparks, MD, USA), 1% yeast extract (Oxoid, Hampshire, UK), 2% glucose (Sbjbio, Nanjing, China) and 0.1% sodium thioglycolate (Sigma Aldrich, St. Louis, MO, USA), and Brucella agar (Hopebio, Qingdao, China) supplemented with hemin (Macklin, Shanghai, China), vitamin K_1_ (Macklin, Shanghai, China) and 5% sheep blood (Sbjbio, Nanjing, China). The *C. perfringens* isolates were cultured at 37°C under anaerobic conditions. The Caco-2 cells (ATCC) was cultured in Minimum Essential Medium (MEM) (Gibco, NY, USA) supplemented with 20% (vol/vol) fetal bovine serum (Gibco) at 37°C with 5% CO_2_.

### Antibacterial activity assays

The minimum inhibitory concentration (MIC) of baicalin-aluminum against *C. perfringens* CVCC2030 was determined using the reference agar dilution method according to the guidelines of the Clinical and Laboratory Standards Institute (CLSI; M11-A8). Briefly, the supplemented Brucella agar plates containing serial two-fold dilutions of various concentrations (ranging from 0 to 800 μg/mL) of baicalin-aluminum were prepared. *C. perfringens* CVCC2030 grown to log-phase was diluted to 1 × 10^5^ colony-forming units (CFUs)/mL. A total of 5 μL cell suspension was inoculated to each plate and incubated in an anaerobic atmosphere for 48 h to observe bacterial growth. The MIC_100_ here was identified as the lowest drug concentration at which bacterial growth remained completely inhibited.

In growth curve assays, overnight cultures of *C. perfringens* CVCC2030 were inoculated into fresh TGY medium supplemented with sub-MICs of baicalin-aluminum (ranging from 0 to 400 μg/mL). Fresh medium adding equivalent concentrations of baicalin-aluminum served as blanks for the subtraction of background turbidity. The cultures were incubated anaerobically at 37°C. Every two hours, the OD_600_ of each culture was measured using a spectrophotometer (BIO-RAD, USA). Growth curves were drawn based on the continuously recorded data to analyze the effect of baicalin-aluminum on the growth of *C. perfringens*.

### Hemolytic activity assay

The hemolytic activity of *C. perfringens* was performed as previously described with some modification (Ali Nasir et al., 2015). *C. perfringens* was cultured in TGY medium with different concentrations of baicalin-aluminum (ranging from 0 to 50 μg/mL) for 5 h. Culture supernatants were collected by centrifugation, sterilized by filtering through 0.22 μm Millex-GP syringe filters (Merck Millipore, Darmstadt, Germany), and serially diluted followed by addition of 2% (V/V) sheep red blood cells (RBCs). RBCs added with an equal volume of sterilized saline and water served as the negative and positive control, respectively. The plate was incubated at 37°C for 1 h. The unlysed cells were precipitated by centrifugation. The hemoglobin released in the cell-free supernatants was measured at OD_540_. The hemolysis rate was expressed as the hemoglobin released in each group divided by that in positive control group. Data were collected from three independent assays.

### Biofilm formation inhibition assay

The crystal violet-based assay was used to quantify the biofilm formation of *C. perfringens* as previously described *in vitro* (Hu et al., 2018). *C. perfringens* was cultured in TGY medium to an OD_600_ of 0.8 and then normalized to 0.1. The bacterial suspensions were inoculated into 200 μL fresh TGY broth (1:100 dilution) containing different concentrations of baicalin-aluminum (ranging from 0 to 50 μg/mL) and placed into a 96-well plate. Fresh TGY broth served as the blank control. Each group was replicated in eight different wells. The plate was subsequently incubated under anaerobic conditions for 72 h. After incubation, the supernatants were aspirated and the plate was gently washed twice with sterile phosphate-buffered saline (PBS) to remove any unbound cells. Then, biofilms were stained with 1% crystal violet for 30 min. The unbound dye was removed from the wells, followed by two additional PBS washes. The bound dye was dissolved by adding 200 μL of methanol for 30 min. The optical density at 570 nm (OD_570_) was measured using a micro-plate reader (Tecan, Switzerland). The assay was performed in three independent experiments.

### Cell viability assay

The cell viability assay was performed using a Cell Counting Kit-8 (CCK-8, Beyotime, Shanghai, China) according to the manufacturer’s instructions as described previously (Zhang et al., 2017). Briefly, the Caco-2 cells were grown in a 96-well plate to obtain monolayer cells. To assess the cytotoxicity of baicalin-aluminum to Caco-2 cells, cells were exposed to various concentrations of baicalin-aluminum (ranging from 0 to 50 μg/mL). To evaluate the effect of baicalin-aluminum on the cytotoxicity of *C. perfringens* to Caco-2 cells, the cells were infected with *C. perfringens* (MOI≈10) and then treated with different concentrations of baicalin-aluminum (ranging from 0 to 50 μg/mL). In the control group, cells were treated without bacteria or drugs (served as 100% cell viability) and in the blank group, no cells were seeded in the wells. The plate was incubated for 6 h at 37°C in a 5% CO_2_ atmosphere and then 10 μL CCK-8 solution was added into each well followed by incubation for 2 h. The OD_450_ was detected using a micro-plate reader. Cell viability was calculated as follows: % cell viability (OD_450_ of treatment group - OD_450_ of blank group)/(OD_450_ of control group - OD_450_ of blank group). The experiments were biologically repeated three times.

### The effect of baicalin-aluminum on the expression of virulence genes

A reverse transcriptase quantitative PCR (RT-qPCR) analysis was carried out to study the impact of baicalin-aluminum on the virulence genes expression of *C. perfringens*. *C. perfringens* was grown in TGY broth with different concentrations of baicalin-aluminum (ranging from 0 to 50 μg/mL) to an OD_600_ of 0.8 at 37°C under anaerobic conditions. Bacterial RNA was isolated using saturated phenol and purified by TRIzol and chloroform as previously described (Li et al., 2013). The purified RNA was used to synthesize cDNA using HiScript qRT SuperMix (Vazyme Biotech). The cDNA amplification was manipulated using AceQ qPCR SYBR Green kit (Vazyme Biotech) in an ABI PRISM 7500 Fast Real-time PCR System. The acquired cycle threshold (CT) of each gene was normalized to the CT of the 16S rRNA. The relative amount of mRNA expression levels was calculated using the 2^−ΔΔCT^ method (Livak and Schmittgen, 2001). The primers utilized in this study are listed in **Table 1**.

**Table 1:** Sequences of primer pairs used for amplification of target and reference genes.

Genes	Strand	Sequences (5’-3’)	Source
*cpa*	Forward	ATGTTACTGCCGTTGATAGCG	This study
	Reverse	TCCTGTTTTAGCAAAACCTCTTG	
*pfoA*	Forward	CTCAGTTGCTGCTGTTCACAAT	This study
	Reverse	TCATCCCAGGCTACTTCAAACT	
*colA*	Forward	GAATGTGGGGACAAGGAGAAT	This study
	Reverse	GTTGAACCTGCAAAGAACTCTG	
*nanI*	Forward	GCTTTTGGAATGCTGGATTAG	This study
	Reverse	TTTGTGGCTCACTCCAAGTCT	
*nanJ*	Forward	TTCTCAAACCCAGACGCAAG	This study
	Reverse	ATTCAGTATATGCCATTCTGCC	
*agrB*	Forward	CATTAGAGGATTCTGAATGTGCT	This study
	Reverse	TTATTCAGTATGGAACTTATGCTCT	
*agrD*	Forward	TTTGGTTCCTCTGGTTGGTG	This study
	Reverse	AAAACTTATTAACATTATTTGCTGC	
*virS*	Forward	TCCTTCAATACAGGCTATGTGAT	This study
	Reverse	TAAAGGACAAGTTAGAAATGGAAT	
*virR*	Forward	CTAAAAGCACGAACTTCATAACC	This study
	Reverse	ACCTTTGAGACAGGAGAGGATC	
16S rRNA	Forward	GGGGGTTTCAACACCTCC	(Matsuda et al., 2009)
	Reverse	GCAAGGGATGTCAAGTGT	
TNF-α	Forward	CCCATCCCTGGTCCGTAAC	(Emami et al., 2019)
	Reverse	ATACGAAGTAAAGGCCGTCCC	
IL-1β	Forward	GCATCAAGGGCTACAAGCTC	(Wang et al., 2019)
	Reverse	CAGGCGGTAGAAGATGAAGC	
IL-6	Forward	CTGTTCGCCTTTCAGACCTACC	(Daneshmand et al., 2022)
	Reverse	GACCACTTCATCGGGATTTATCA	
GAPDH	Forward	TTGTCTCCTGTGACTTCAATGGTG	(Daneshmand et al., 2022)
	Reverse	ACGGTTGCTGTATCCAAACTCAT	

### Chicken necrotic enteritis model

The animal experiment was carried out at the Wuhan Polytechnic University, China. A total of 60 1-day-old healthy Ross 308 broilers with similar body weights were purchased from Zhengkang Poultry Co., Ltd, Jingzhou, China. All the broilers were weighted and randomly divided into six groups, including a control group (CON), a *C. perfringens* infection group (CP), a lincomycin group (LIN), and three baicalin-aluminum (BAl) treatment groups (BAl1, BAl2, BAl3), with 10 broilers in each group. All broilers were allowed to receive water and diet ad libitum. The temperature was maintained at 33°C for the first 5 days and then gradually decreased by 3°C a week until the final temperature reached 26°C. The relative humidity was about 65% and lighting was provided with a 23:1 h light:dark cycle during the entire experiment. The necrotic enteritis model was constructed by using *C. perfringens* CVCC 2030 strain as previously described with some modifications (Zheng et al., 2021). Briefly, from day 14 to day 20, broilers except those in the control group were orally dosed once a day with 1 mL *C. perfringens* CVCC2030 suspension with approximately 5×10^8^ CFU, and broilers in the control group received 1 mL sterile medium. After infection, broilers in the control group and infection group were fed with a basic diet; broilers in the BAl1, BAl2, and BAl3 were fed a basic diet supplemented with 1 g/kg, 2 g/kg and 4 g/kg baicalin-aluminum, respectively; broilers in the LIN group were given 40 mg/kg lincomycin in the basic diet. In this period, clinical signs were recorded in different groups in detail. On days 14 and 21, the broilers were feed-deprived for 8 h and then weighed for the calculation of body weight gain. On day 21, all animals were euthanized.

The lesion scoring in the jejunum and ileum of each broiler was conducted as previously described lesion score system that scaled from 0 to 6 (Keyburn et al., 2006). The jejunal and ileal tissue were collected for RNA analysis, H&E staining and histopathology analysis. Total RNA of jejunal and ileal tissue was extracted by using RNeasy Mini Kit (Qiagen GmbH, Hilden, Germany) according to the manufacturer’s instructions. The cDNA was prepared and mRNA levels of proinflammatory genes (TNF-α, IL-1β, IL-6, IL-10) were determined by qPCR as described above and normalized to GAPDH. The primers utilized are listed in **Table 1**.

The genomic DNA was extracted from ileal digesta samples by using the QiagenQIAamp DNA Stool Mini Kit (Qiagen, Valencia, CA, USA). The relative quantity of intestinal flora in the ileal digesta were quantified using specific 16S rDNA primers of *C. perfringens*, *Escherichia coli* and *Lactobacillus* group (including *Leuconostoc*, *Pediococcus*, *Aerococcus* and *Weissella* but not *Enterococcus* or *Streptococcus*)by qPCR as previously described (Li et al., 2022). The total bacteria (16s rDNA) were used as the reference. The primers targeting the bacteria were as follows: *C. perfringens*: F-AAAGATGGCATCATCATTCAAC and R-TACCGTCATTATCTTCCCCAAA (Wang et al., 1994); *Lactobacillus*: F-AGCAGTAGGGAATCTTCCA and R-CACCGCTACACATGGAG (Walter et al., 2001; Heilig et al., 2002); *E. coli*: F-GTTAATACCTTTGCTCATTGA and R-ACCAGGGTATCTAATCCTGT (Li et al., 2022); total bacteria: F-ACTCCTACGGGAGGCAG CAGT and R-GTATTACCGCGGCTGCTGGCAC (Li et al., 2022).

### Statistical analyses

Data were collected and analyzed using GraphPad Prism version 6 software. The data of lesion scores was analyzed by the Mann-Whitney test and showed in the median and interquartile range. The data other than the lesion scores was analyzed using Student’s two-tailed *t*-test and presented as mean ± standard deviation (SD). *p < 0.05 and **p < 0.01 were considered to be statistically significant.

## Results

### Antibacterial activity of baicalin-aluminum against *C. perfringens*

No obvious difference in colony formations was observed between the positive group (0 μg/mL) and the baicalin-aluminum-treated group (800 μg/ml) after 48 h at 37°C, while no visible bacterial growth was seen in the negative control group, indicating that the MIC_100_ of baicalin-aluminum against *C. perfringens* was greater than 800 μg/mL (**Figure 1B**). Higher concentrations of baicalin-aluminum were not explored because of the solubility. The growth curves indicated that bacterial growth was inhibited within 12 h by the baicalin-aluminum at a concentration above 100 μg/mL, while no significant effect was observed in the growth of *C. perfringens* treated with baicalin-aluminum at a concentration below 50 μg/mL (**Figure 1C**).

### Baicalin-aluminum decreases the hemolytic activity of *C. perfringens*

**Figure 2:**
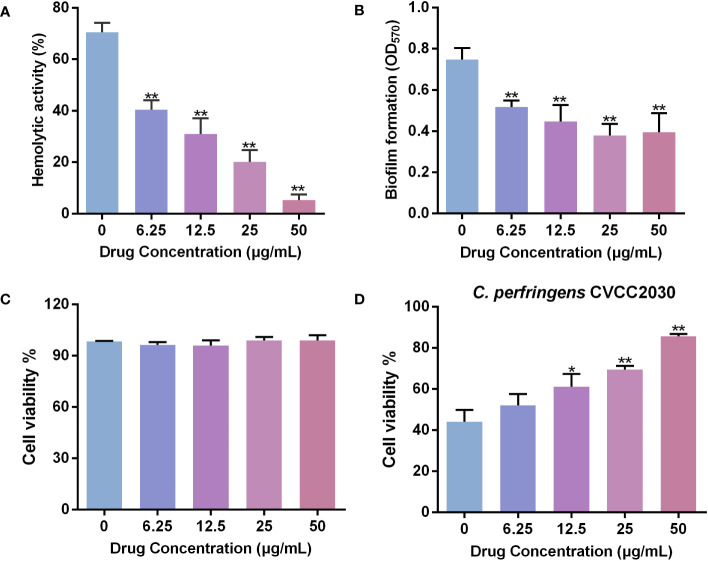
Baicalin-aluminum inhibits the hemolysis, biofilm formation and cytotoxicity to Caco-2 cells of *C. perfringens*. **(A)** Inhibition of *C. perfringens*-induced hemolysis (%) by baicalin-aluminum. Hemolysis assays were performed by co-incubating 2% sheep RBCs with the culture supernatants of *C. perfringens* treated with various concentrations of baicalin-aluminum. The 2% RBCs added with an equal volume of water served as the positive control. The hemoglobin released in the cell-free supernatants due to the lysis of RBCs was measured at OD_540_. The hemolysis rate of each group was calculated by comparison the hemoglobin released with the positive control group. **(B)** Biofilm formation of *C. perfringens* treated with baicalin-aluminum. The biofilms formed of *C. perfringens* treated with or without baicalin-aluminum were stained with 1% crystal violet, dissolved with methanol and measured at OD_570_. **(C)** Caco-2 cells viability in the presence of baicalin-aluminum. **(D)** Cell viability of Caco-2 cells infected by *C. perfringens* followed by treated with baicalin-aluminum. Data are presented as the mean ± SD from three biological replicates. *p < 0.05 or **p < 0.01 indicates a significant difference between the groups with or without baicalin-aluminum treatment.

Studies have shown that certain toxins produced by *C. perfringens*, including CPA, and PFO, can induce hemolysis of red blood cells (Liu et al., 2020). Here, the effect of baicalin-aluminum on the hemolytic activity of *C. perfringens* was determined. As shown in **Figure 2A**, the hemolysis rate of *C. perfringens* CVCC2030 was significantly decreased by 42.61% after incubated with baicalin-aluminum at a concentration of 6.25 μg/mL compared with that in non-drug-exposed group. The data indicated that baicalin-aluminum could inhibit the production or activity of *C. perfringens* hemolytic toxins.

### Baicalin-aluminum inhibits the biofilm formation of *C. perfringens*

The biofilm-formation ability of *C. perfringens* incubated with baicalin-aluminum at a concentration of 6.25 μg/mL was significantly decreased by 30.85% compared to that without baicalin-aluminum (**Figure 2B**). The result indicated that baicalin-aluminum was conducive to diminish the biofilm formation of *C. perfringens in vitro*.

### Baicalin-aluminum decreases the cytotoxicity of *C. perfringens* to Caco-2 cells

The result of CCK-8 assay showed that baicalin-aluminum (ranging from 6.25 to 50 µg/mL) had no cytotoxicity to Caco-2 cells (**Figure 2C**). Additionally, the cell viability increased by 17.15% in the coculture system of Caco-2 cells with *C. perfringens* CVCC2030 when treated with 12.5 μg/mL baicalin-aluminum (**Figure 2D**). The result suggested that baicalin-aluminum treatment could protect Caco-2 cells from injuries mediated by *C. perfringens*.

### Baicalin-aluminum is involved in the virulence factors expression of *C. perfringens*

**Figure 3:**
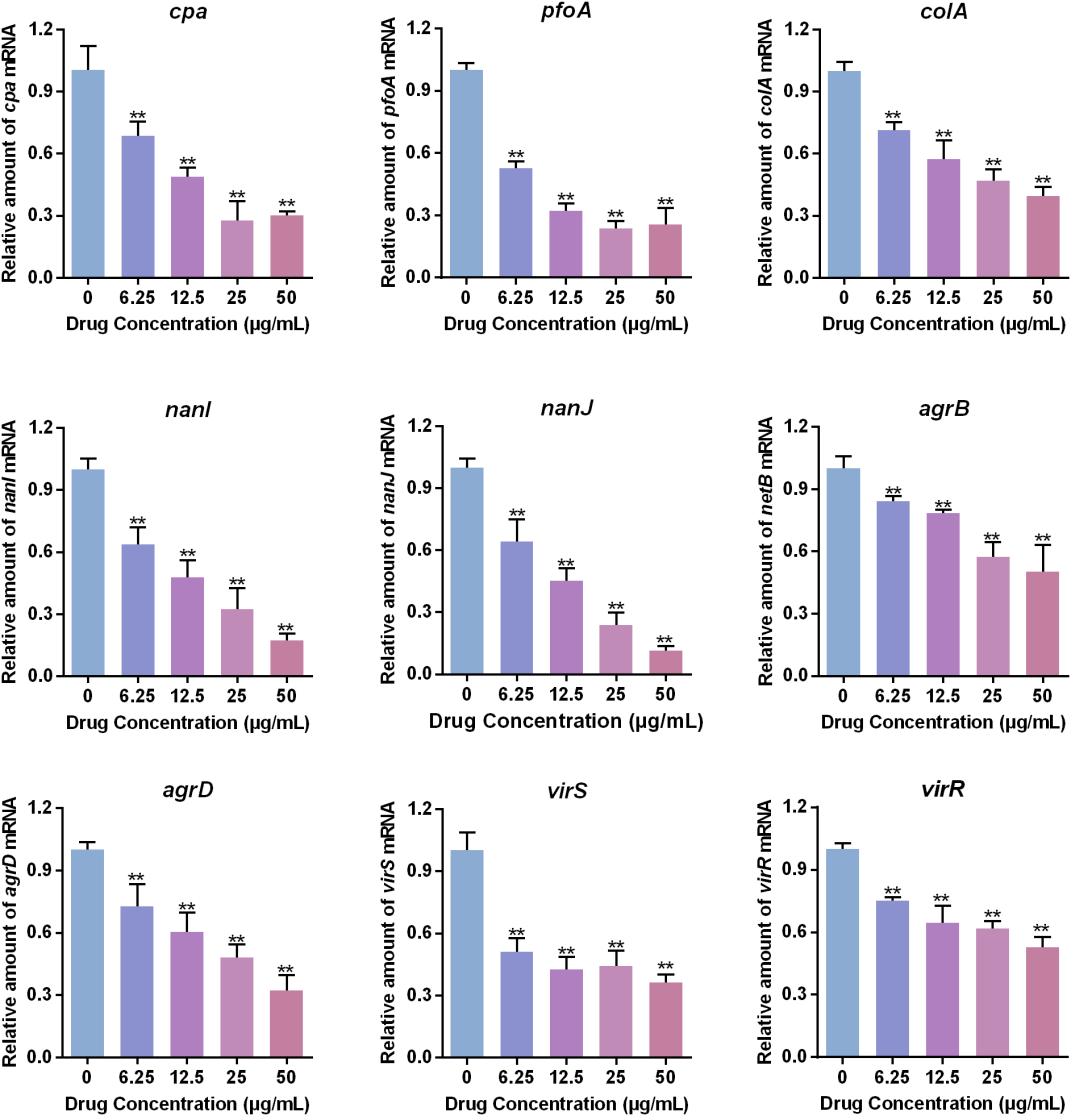
Effects of baicalin-aluminum on the virulence factors expression of *C. perfringens*. *C. perfringens* CVCC2030 treated with various concentrations of baicalin-aluminum were cultured to OD_600_ of 0.8 at 37°C under anaerobic conditions. The mRNA levels of *cpa*, *pfoA*, *colA*, *nanI*, *nanJ*, *agrB*, *agrD*, *virS*, and *virR* were determined by RT-qPCR. For each sample, the acquired cycle threshold (CT) was normalized to the CT of 16S rRNA, and the ΔCT of each gene in baicalin-aluminum-treatment group was normalized to that in drug-free group. Relative amounts in transcription level were calculated using the 2^−ΔΔCT^ method. Data are presented as the mean ± SD of three independent experiments, with each experiment being consisting of three replicates. **p < 0.01 indicates a significant difference between the groups with or without baicalin-aluminum treatment.

To determine whether baicalin-aluminum affects the virulence factors expression of *C. perfringens*, RT-qRCR analysis was performed. The results in **Figure 3** showed that the toxins encoding genes (*cpa*, *pfoA*, *colA*, *nanI, nanJ*) were downregulated when *C. perfringens* CVCC2030 was treated with baicalin-aluminum. In addition, the mRNA levels of the genes involving in regulating toxins production, including Agr QS system and VirS/R two-component regulatory system (TCRS) associated genes (*agrB*/*agrD*, *virS*/*virR*) were decreased when *C. perfringens* was exposed to baicalin-aluminum. The data indicated that baicalin-aluminum was able to inhibit virulence factors expression of *C. perfringens* in a dose-dependent manner.

### Protective effect of baicalin-aluminum against *C. perfringens* infection

To evaluate the *in vivo* effect of baicalin-aluminum in the development of necrotic enteritis, broilers were orally infected with *C. perfringens* and given baicalin-aluminum in the basic diet.

**Figure 4:**
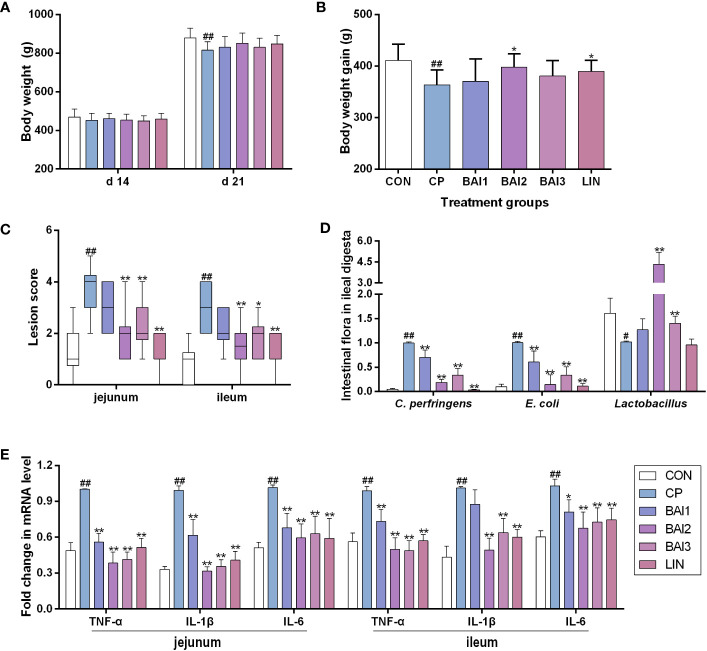
Effects of baicalin-aluminum treatment on the body weight, body weight gain, intestinal lesion score, cytokines expression, and the number of bacteria in the ileal digesta of broiler chickens challenged with *C. perfringens*. The body weight **(A)** and body weight gain **(B)** were calculated during the infection and treatment period. The data of the intestinal lesion score is expressed in the median and interquartile range **(C)**. The levels of some bacteria in ileal digesta **(D)** and cytokines (TNF-α, IL-1β, IL-6) expression in the jejunal and ileal tissues **(E)** were measured by qRT-PCR. Values in **(A, B, D, E)** were presented as the mean ± SD. CON, the control group; CP, *C. perfringens* challenged group; BAl1, BAl2 and BAl3, 1kg basic diet supplemented with 1 g, 2 g, and 4 g baicalin-aluminum, respectively; LIN, 1kg basic diet supplemented with 40 mg lincomycin. #*p* < 0.05 vs. CON, ##*p* < 0.01 vs. CON, *p < 0.05 vs. CP, **p < 0.01 vs. CP.

*C. perfringens* challenge decreased the body weight and body weight gain of broiler chickens during the infection period, while dietary baicalin-aluminum (2 g/kg) heightened the weight gain of broilers (**Figures 4A, B**).

No obvious necrotic lesions were seen in the jejunum and ileum of broilers in the control group that were not challenged with *C. perfringens.* The infection group challenged with *C. perfringens* CVCC 2030 displayed increased necrotic lesion scores both in the jejunum and ileum, while only mild pathological damage and a decreased lesion score was detected in the baicalin-aluminum treatment group compared with the infection group (**Figure 4C**).

As shown in **Figure 4D**, the relative abundance of *C. perfringens* and *E. coli* in the ileal digesta were raised by the *C. perfringens* challenge, while baicalin-aluminum treatment decreased them. Compared with the control group, the relative number of *Lactobacillus* in the ileal digesta was decreased, and expectedly, dietary baicalin-aluminum (2 g/kg, 4 g/kg) upregulated it. It was suggested that baicalin-aluminum exerted beneficial effects in *C. perfringens*-challenged broilers.

The mRNA levels of IL-1β, IL-6 and TNF-α in the jejunal and ileal tissues were increased after *C. perfringens* challenge relative to that in the control group (**Figure 4C**). Baicalin-aluminum significantly decreased the cytokines levels of IL-1β, IL-6, and TNF-α mRNAs (**Figure 4E**).

**Figure 5:**
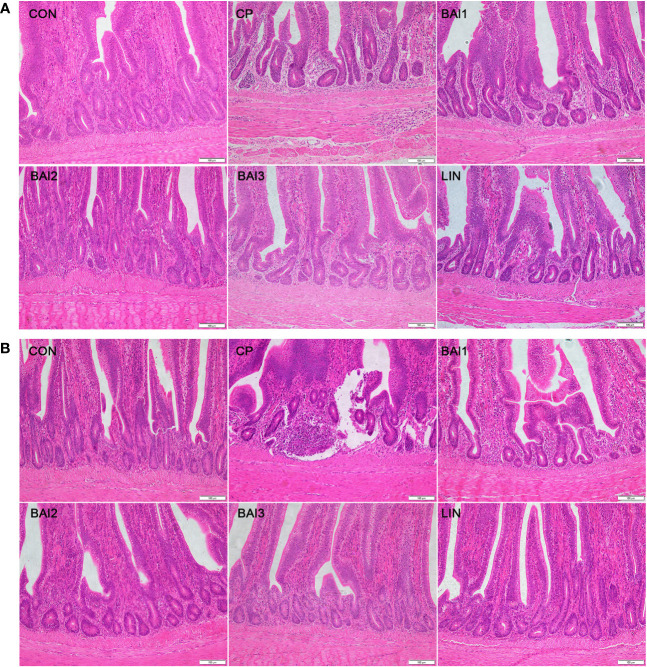
Comparison of microscopic pathological changes in the jejunum **(A)** and ileum **(B)** after treatment with baicalin-aluminum. Histopathology was performed using an upright microscope at 200× magnification.

Additionally, inflammatory cell aggregation was detected in the jejunum or ileum of the infection group (**Figure 5**). However, only minor histopathological damage was detected in the jejunum or ileum of the broilers in the baicalin-aluminum and lincomycin treatment groups (**Figures 5**).

## Discussion

Necrotic enteritis caused by *C. perfringens* continues to pose a significant threat and challenge to the poultry industry. Traditional antibiotics therapeutic strategy by stopping bacterial growth puts tremendous selective pressure on pathogen and drives the development of drug resistance. Since virulence factors are usually not indispensable components of bacterial survival, interference with them will exert less pressure on the selection of pathogens and is less susceptible to bacterial resistance (Dehbanipour and Ghalavand, 2022). Therefore, anti-virulence therapy through blocking virulence factors or virulence-associated processes has been shown to be a new alternative therapeutic strategy for bacterial infection. Numerous studies indicated that some plant-derived bioactive compounds were able to control bacterial infection via targeting virulence factors (Liu et al., 2020; Hou et al., 2023; Martínez et al., 2023). It is found that tectorigenin, an isoflavone extracted from the rhizome of the Chinese herb *Belamcanda chinensis* (L.) DC, displayed no observable antibacterial activity against *C. perfringens*, but it significantly inhibited the gliding motility, biofilm formation and adherence to Caco-2 cells of the bacteria by suppressing type IV pilus-associated genes expression (Liu et al., 2019). Baicalin was able to attenuate the *in vivo* pathogenicity of *Pseudomonas aeruginosa* by diminishing a good many of important virulence factors and cellular toxicity (Zhang et al., 2021). In order to handle with *C. perfringens* infections, the effects of baicalin-aluminum on the virulence factors expression and the *in vivo* therapeutic effect on NE were tested in the present study. It is found that baicalin-aluminum was able to inhibit hemolytic activity, biofilm formation, cytotoxicity, and expression of QS-related virulence genes of *C. perfringens* without inhibiting bacterial growth. In addition, baicalin-aluminum treatment significantly lightened the intestinal histological damage, altered the quantity of intestinal flora, as well as inhibited the levels of pro-inflammatory cytokines (TNF-α, IL-1β, IL-6) of broiler chicken challenged with *C. perfringens*.

The virulence of *C. perfringens* is largely attributable to the production of toxins and extracellular enzymes, which are required for the pathogenesis of necrotic enteritis. CPA possesses phospholipase C, sphingomyelinase and hemolytic activities, it can lead to the lysis and death of intestinal epithelial cells by destroying the cell membrane structure. The amount of CPA detected in the intestine of broiler chickens with necrotic enteritis is closely related to the intestinal lesions (Coursodon et al., 2010). PFO is a pore-forming toxin that bind to cell membranes and oligomerize to a pore complex to lyse cells, it has a synergistic effect with other toxins in the pathogenic process of *C. perfringens* (Fernandez-Miyakawa et al., 2008; Verherstraeten et al., 2013). Besides, some enzymes such as sialidases, especially NanI, can enhance the production and cytotoxicity of toxins associated with intestinal infections, facilitate the adhesion and colonization of *C. perfringens* in the gut, and produce substrates used for bacterial growth and survival, emerging as potential pathogenic factors during *C. perfringens* intestinal infections (Wang, 2019). Several collagenolytic enzymes including ColA were expressed at high levels in the intestinal tissues of broilers challenged with *C. perfringens* isolated from field cases of NE (Olkowski et al., 2008). Importantly, we found that the *cpa*, *pfoA*, *colA*, *nanI* and *nanJ* genes were significantly downregulated in *C. perfringens* after treatment with baicalin-aluminum. Moreover, CPA and PFO are considered as hemolytic toxins and are responsible for the cytotoxicity to mammal cells (Liu et al., 2020). Here, we found that baicalin-aluminum could diminish hemolysis and protect Caco-2 cells from cytotoxicity mediated by *C. perfringens in vitro*. Therefore, baicalin-aluminum might be able to attenuate the virulence of *C. perfringens* by inhibiting the expression and production of toxins without stopping bacterial growth.

QS system is largely responsible for the regulation of virulence genes expression and other group behaviors such as biofilm formation in many bacteria (Vashistha et al., 2023). The Agr system in *C. perfringens* is considered to be the most important QS system of the bacterium for virulence, which plays an important role in the pathogenesis of necrotic enteritis in chickens (Yu et al., 2017). This system generates AIP to signal a VirS/R TCRS and they acted synergistically to regulate the production of several *C. perfringens* toxins and enzymes, including PFO, CPA, ColA, NanI and NanJ (Ohtani and Shimizu, 2016; Fathima and Hakeem, 2022). Therefore, targeting QS system will be a hopeful anti-virulence approach. This study demonstrated that the mRNA levels of Agr QS system and VirS/R TCRS associated genes (*agrB*, *agrD*, *virS*, *virR*) were decreased by baicalin-aluminum, suggesting that baicalin-aluminum was able to inhibit QS system. Moreover, scanning electron microscope (SEM) and transmission electron microscope (TEM) observation indicated that the cell morphology and ultrastructural structure of *C. perfringens* treated with 50 µg/mL baicalin-aluminum did not show any obvious change compared to that of non-baicalin-aluminum-exposed cells (**Supplementary Figure 1**), suggesting that baicalin-aluminum might achieve antibacterial purposes not by inhibiting the synthesis of bacterial cell walls, but by inhibiting target proteins or DNA. We speculate that baicalin-aluminum inhibits the expression of *C. perfringens* virulence factors by interfering with the Agr QS system, which requires further study to be verified. Additionally, the Agr QS system is also intimately linked to the biofilm formation in *C. perfringens* by increasing the levels of certain toxins including CPA and PFO required to build biofilms, which could be important during infections (Vidal et al., 2015). In this regard, whether the reduction of biofilms formed by *C. perfringens* exposed to baicalin-aluminum is correlated with the inhibition of Agr QS system or toxins production needs our further investigation.

Since the virulence factors inhibited by baicalin-aluminum were involved in the disease pathogenesis of *C. perfringens*-mediated infection, the *in vivo* effects of baicalin-aluminum on the development of necrotic enteritis were evaluated. In the present study, broilers in the control group showed no obvious necrotic lesions in the jejunum and ileum. Severe pathological damage and increased intestine lesion score were observed after *C. perfringens* challenge, which also was demonstrated by Kumar et al. (Kumar et al., 2021; Zheng et al., 2021). In contrast, broilers treated with baicalin-aluminum at three levels and lincomycin all displayed only mild pathological damage and decreased lesion scores compared with the infection group. Cytokines are important mediators of inflammation, immunity, and the pathological damage that occurs during the disease processes (Liu et al., 2021). It is known that pro-inflammatory cytokines such as TNF-α, IL-6, and IL-1β are closely related to the inflammatory response and cell death of intestinal epithelial, could be produced after stimulation by pathogens (Wu et al., 2021; Daneshmand et al., 2022). In this report, the mRNA levels of IL-1β, IL-6, and TNF-α were significantly upregulated in the intestine tissues over *C. perfringens* challenge periods. However, the supplementation of baicalin-aluminum significantly reduced the expression of the cytokines, and the levels of the cytokines tended to downregulate linearly and quadratically as the baicalin-aluminum level increased and were all the lowest at 2 g/kg in the diet. In addition, we discovered that lincomycin also markedly downregulated the cytokines (IL-1β, IL-6, and TNF-α) express levels, which is consistent with the results previous reported (Wang et al., 2015). Excessive production of pro-inflammatory cytokines could result in the occurrence of chronic inflammation in the gut and intestine lesion (Leppkes et al., 2014). Inflammatory cell aggregation was detected in the jejunum or ileum of the infection group, while only minor histopathological damage was detected in the baicalin-aluminum and lincomycin treatment groups. The results demonstrated that the application of baicalin-aluminum could effectively alleviate intestinal pathological damage in broilers.

Additionally, the composition and quantity of gut microbes is a key factor in maintaining integrity of the intestinal structure, promoting maturation of the immune system, and modulating host immune responses (Obianwuna et al., 2023). Studies indicated that intestinal flora would be shaped by *C. perfringens* challenge, resulting in a reduction of beneficial bacteria and an increase of pathogenic bacteria (Lu et al., 2021). Consistent with this, our study showed that the relative abundance of *C. perfringens* and *E. coli* was raised and that of *Lactobacillus* in the ileal digesta was reduced after *C. perfringens* challenge. Expectedly, baicalin-aluminum was able to alleviate the upregulation of *C. perfringens* and *E. coli* and improve the relative abundance of *Lactobacillus.* This finding is similar with a previous report in which the broiler gut microbiome composition was modified by baicalin-aluminum with enhanced abundances of the probiotic bacteria (Guo et al., 2021). The data suggested baicalin-aluminum had a potential role in improving intestinal environment. Finally, the growth performance has been reported to be negatively affected by the *C. perfringens* challenge (Tang et al., 2022). Consistent with this, the body weight gain of broiler chickens was reduced after *C. perfringens* infection in this study. Notably, baicalin-aluminum weakened the adverse effects of *C. perfringens* on the weight gain, which may be explained by alleviating the intestinal pathological damage and improving the microbiota structure.

In summary, our results reveal that baicalin-aluminum has inhibitory effect on the virulence genes expression and virulence-associated traits of *C. perfringens in vitro*, thereby conferring protection against *C. perfringens* infection in a broiler necrotic enteritis model. The data suggests that baicalin-aluminum exhibits potential as a novel anti-virulence drug for the prevention and control of *C. perfringens* infection.

## Data availability statement

The original contributions presented in the study are included in the article/**Supplementary Material**. Further inquiries can be directed to the corresponding author.

## Ethics statement

The animal study was approved by Institutional Animal Care and Use Committee of Wuhan Polytechnic University, China. The study was conducted in accordance with the local legislation and institutional requirements.

## Author contributions

JL and SW carried out most of the experiments described in the manuscript. JL wrote the article. HZ, CY, SF, YL, and TL participated in the collection and analysis of the data. YQ provided expertise and conceived the study. All authors contributed to the article and approved the submitted version.
