# Supplementary material for: Baicalin-aluminum alleviates necrotic enteritis in broiler chickens by inhibiting virulence factors expression of Clostridium perfringens
Source: Front Cell Infect Microbiol. 2023 Sep 25;13:1243819. doi: 10.3389/fcimb.2023.1243819 (PMC10561085; doi:10.3389/fcimb.2023.1243819)
Supplement: Supplementary file 1 [file DataSheet_1.pdf]

Supplementary Material

Baicalin-aluminum alleviates necrotic enteritis in broilers by inhibiting virulence factors expression of *Clostridium perfringens*

Jin Liu[†], Shuangqi Wu [†], Honghao Zhao, Chun Ye, Shulin Fu, Yu Liu, Ting Liu, and Yinsheng Qiu[*]

[†] These authors contributed equally to the study.

[*] **Correspondence:** Yinsheng Qiu: qiuyinsheng6405@aliyun.com

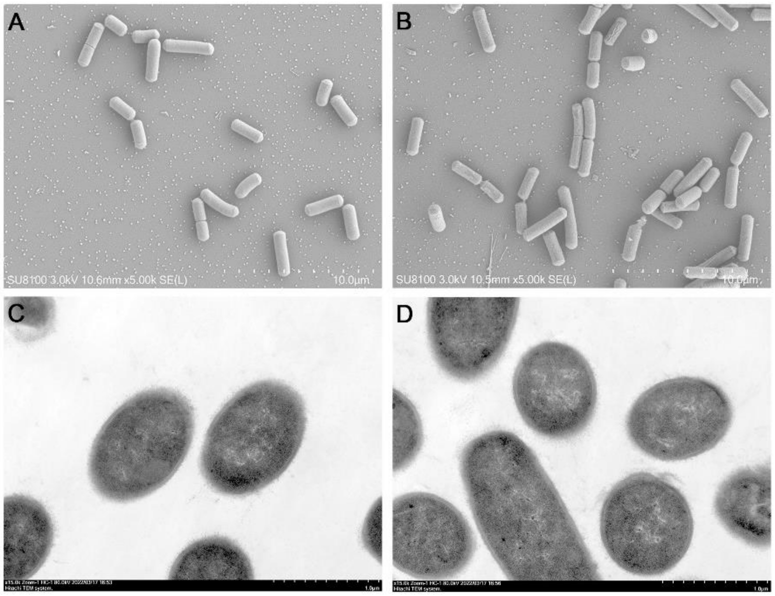

Supplementary Figure 1. TEM and SEM observation. *C. perfringens* DK2 cultured without drugs were observed under TEM (A) and SEM (C). *C. perfringens* DK2 cultured with 50 µg/mL baicalin-aluminum for 6 h at 37℃ under anaerobic conditions were observed under TEM (B) and SEM (D).
